# CassavaMap, a fine-resolution disaggregation of cassava production and harvested area in Africa in 2014

**DOI:** 10.1038/s41597-020-0501-z

**Published:** 2020-05-27

**Authors:** Anna Maria Szyniszewska

**Affiliations:** Department of Plant Sciences, University of Cambridge, Cambridge, UK

**Keywords:** Agroecology, Biogeography, Ecological modelling

## Abstract

Cassava, the third main source of carbohydrates in Africa, provides daily nutrition for over 700 million people on the continent and a vital source of income for subsistence farmers. Despite its importance, our knowledge of the heterogeneity of its distribution in the landscape is limited and outdated. Information on cassava production and harvested area are typically available on aggregated administrative unit level with highly variable temporal range of records, often over a decade old, and represented on a coarse ~10 km by 10 km grid. Here, cassava production and harvested area administrative unit level data for 32 countries are standardised to 2014 FAO reported levels and disaggregated based on the distribution of the rural population in 2014. The grid obtained represents a significant improvement on the previous studies in terms of both spatial resolution (~1 km by 1 km) and temporal accuracy. Enhanced representation of cassava production and harvested area in Africa is an essential resource for policy making as well as designing strategies to manage its main pathogens.

## Abstract (summary)

**Table:** 

Measurement(s)	plantation • total biomass yield
Technology Type(s)	digital curation
Factor Type(s)	geographic location • rural population • crop production statistics
Sample Characteristic - Organism	Manihot esculenta
Sample Characteristic - Environment	area of cropland • cultivated environment • agricultural field
Sample Characteristic - Location	Africa

Machine-accessible metadata file describing the reported data: 10.6084/m9.figshare.12279938

## Background & Summary

Cassava (*Manihot esculenta* Krantz) is a root crop grown in the tropics. It is a rich source of calories and provides several important micronutrients^1,2^. Cassava is grown mainly for subsistence and direct consumption, but has a growing market of processing opportunities^3^. Currently, its production in sub-Saharan Africa is drastically hindered by several pests and diseases. The most devastating of these are the virus diseases – cassava mosaic disease (CMD), and cassava brown streak disease (CBSD)^4–7^.

Considerable resources have been invested by various agricultural development donors in surveillance activities for those diseases, as well as in the development and deployment of control strategies, including clean seed and resistant varieties^8–10^. In all of these activities, a reliable representation of the distribution of cassava cultivation is an essential resource for planning. However, our knowledge about the spatial distribution of cassava from currently existing published studies or output from governments of international agencies such as FAO is outdated, and often represented only by national or sub-national level statistics for cassava production or harvested area^11^.

There are two resources known to the author that represent the results of the modelling of the distribution of cassava production and harvested area based on agriculture census data and disaggregating it to grid. The first model, available via the CGIAR Research Program on Roots, Tubers and Bananas (RTB)^12^, provides information on the spatial distribution of numerous crops including cassava. More specifically, it provides information on the spatial distribution of cassava harvested area, yield gap, potential yield and so called cassava suitability index with a spatial resolution at the equator of approximately 10 km by 10 km. These maps were obtained by disaggregating statistics on harvested areas and yields of 175 crops among cropland areas represented by two different land cover classification sources: MODIS and GLC2000, and represent production statistics for ca. 2000. The second source of information on the distribution of crops is the Spatial Production Allocation Model (MapSPAM)^13–15^. MapSPAM uses a variety of inputs (crop production statistics, irrigated land, croplands, rural population and crop biophysical suitability index) to disaggregate the production of 42 crops including cassava simultaneously using an entropy-based data-fusion approach^14^. At the time of writing this paper, the newest version of MapSPAM was representing crop production statistics for 2000, 2005 and 2010, depending on the version, at ~10 km by 10 km resolution globally or ~1 km by 1 km resolution on a country level for some countries. This model used data on harvested area to estimate the areas of cropland in each administrative unit.

Mapping cassava on a continental scale based on satellite imagery remains a major challenge. Cassava in sub-Saharan Africa dominates in small-holder farming systems with field sizes most frequently less than 1000 m^2^, which are often intercropped^16,17^. Field rotation, flexible harvesting times and variable planting seasons means there is no strongly defined pattern of field maturation in many areas. In addition, the variability of cultivars and (most importantly) agro-ecological zones means the cassava spectral signal and texture pattern on satellite imageries varies greatly. The combination of these challenges motivated this work. The objective was to find an alternative approach of estimating cassava production and harvested area density based on available proxy variables.

**Fig. 1:**
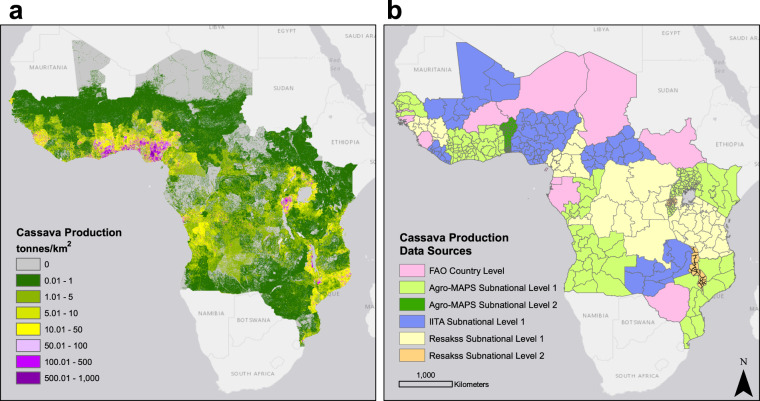
Disaggregated cassava production in sub-Saharan Africa and associated data sources. Density of cassava production layer (tonnes per approx. 1 km^2^) (**a**). Cassava production data sources and administrative resolution of production census data in each of the countries (**b**): FAO (fao.org/faostat), Resakss (resakss.org), Agro-MAPS (kids.fao.org/agromaps/) and IITA (personal correspondence with the International Institute of Tropical Agriculture in Ibadan, Nigeria). Basemap source: Esri, DeLorme, HERE, MapmyIndia.

**Fig. 2:**
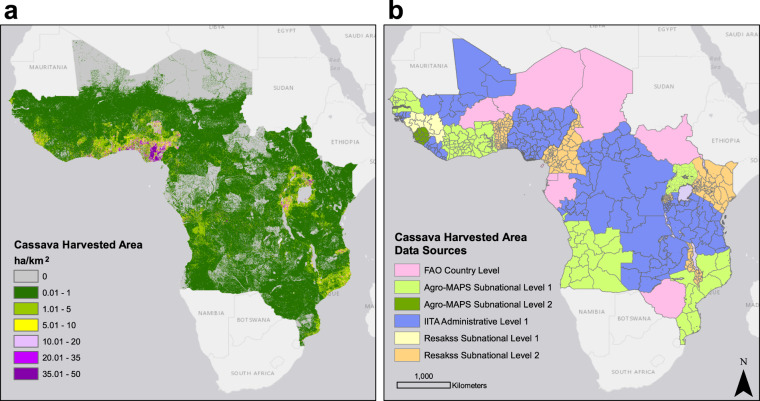
Disaggregated cassava harvested area in sub-Saharan Africa and associated data sources. Density of cassava harvested area layer (hectares per approx. 1 km^2^) (**a**) and harvested area data sources as well as administrative resolution of data in each of the countries (**b**): FAO (fao.org/faostat), Resakss (resakss.org), Agro-MAPS (kids.fao.org/agromaps/) and IITA (personal correspondence with the International Institute of Tropical Agriculture in Ibadan, Nigeria). Basemap source: Esri, DeLorme, HERE, MapmyIndia.

Previous studies have shown that the distribution of non-urban population is an important predictor of the density of cassava in the landscape in sub-Saharan Africa, and it’s relative importance varies depending on regional cultural preferences^18–21^. The crop is adaptable to various environmental conditions and there are no limiting factors in terms of its distribution like temperature or rainfall in the majority of the study region. Unlike rice and maize, it is highly drought resistant and can be grown on marginal soils^18^. Due to the limited number of non-environmental ancillary covariates that are likely to influence the preference of cassava planting in sub-Saharan Africa like cultural preferences or poverty, the distribution of rural population was assumed to be the main driver of cassava density in the landscape and the relationship between population number and cassava production and harvested area density linear up to defined maximum threshold. Available cassava production and harvested area data obtained from various existing sources including FAO, ReSAKSS, and International Institute of Tropical Agriculture (IITA) communication were used as proxies representing regional importance of the crop. The administrative level cassava production and harvested area data were disaggregated into small spatial units (pixels) at approximately 30′′ × 30′′ (~1 × 1 km) resolution, between rural population data obtained from LandScan 2014. The two layers obtained provide the finest up-to-date representation of cassava distribution in the landscape in 30 countries of sub-Saharan Africa currently available (Figs. 1 and 2).

## Methods

### Rural population

Representation of human population density was used to redistribute data on cassava production per province or district in 30 countries of West, central, East and southern Africa. Data were obtained from the LandScan 2014^22^ raster population density layer developed as part of the Oak Ridge National Laboratory (ORNL) Global Population Project at a resolution of approximately at ~1 km by 1 km (~30′′ by 30′′). The LandScan model uses country sub-national census data and combines it with additional datasets including land cover, roads, urban and rural locations to redistribute the populations according to a weighting scheme. Populations living in densely populated urban areas are unlikely to grow cassava and thus, areas with a population density higher than 5,000 inhabitants per 1 km^2^ were masked out and deemed unsuitable for cassava farming.

### Cassava production and harvested area data

Sub-national data about cassava production and harvested area per administrative unit were obtained and compiled from several sources: FAO Agro-MAPS (www.kids.fao.org/agromaps), ReSAKSS (www.resakss.org) and personal communication with the International Institute of Tropical Agriculture (IITA). These sources were later compared according to the administrative resolution, year of data records and amount of missing data. Based on these three criteria, the preferred data source was manually identified to strike the best balance most recent records, fine resolution and finally considering number of missing records (Online-only Tables 1 and 2). For some countries no sub-national statistics on cassava production or harvested area were available. In those instances, country level FAO estimates (www.fao.org/faostat) for 2014 were used for cassava production and harvested area estimates (Online-only Tables 3 and 4). Collated data represented statistics from various years, as agricultural censuses are taken rarely, often every 10 years or even less frequently. Country-level FAO cassava production and harvested area estimates from 2014 were used to standardise values in the administrative units across each country so the totals represented those of the 2014 national-level estimates.

### Administrative units

Due to frequent changes in administrative divisions in various African countries, polygon shapefiles provided with respective sources of cassava production statistics were used to represent the extent of administrative zones. Administrative division shapefiles for Uganda provided by FAO Agro-MAPS were unfit for use due to the corrupted topology of the layer and the lack of a match between the unique identifiers and names of the districts listed in the production data file. In this case, the country administrative shapefile for the first level of administrative division was downloaded from GADM (www.gadm.org). Unions between districts that have been historically merged were applied in order to link them correctly to the Agro-MAPS statistics file.

### Missing data

In multiple subnational administrative units, production, or harvested area data respectively were missing (Online-only Tables 3 and 4). Based upon personal communication with country experts it was established that cassava is grown in those areas. In those areas cassava per capita production/harvested area values were calculated individually based either on the average value of all administrative units from the same country adjacent to the unit with missing data or based on values in districts representing the same or similar agro-ecological zones (Online-only Table 5).

The Ugandan country outline shapefile obtained from GADM had relatively small areas that lacked alignment with neighbouring Kenya and Democratic Republic of Congo (DRC) shapefiles. It resulted in two areas containing a proportion of pixels with missing values within the bounding boxes defined by the following sets of coordinates: 35.797°E, 4.146°N, 33.963°W, 1.569°S and (30.993°E, 3.463°N, 30.742°W, 2.514°S. Missing value pixels in these bounding boxes were replaced with a constant value obtained from the range of values in neighbouring pixels (0.02 for production and harvested area per capita 2014 adjusted values).

### Disaggregation

For each administrative unit, cassava production in tonnes and harvested area in hectares per capita values adjusted for 2014 FAO reported country totals (*Prod_percapita_2014* and *HA_percapita_2014*) were calculated. They were obtained by dividing the adjusted production total (*Admin_unit_prod2014* and *Admin_unit_ha2014*) (Eqs. 1 and 2) by the rural population (*Admin_unit_pop2014*) (areas at or below 5,000 inhabitants per pixel) in each administrative unit (Eqs. 3 and 4).

1 $$Admin\_unit\_prod\_2014=\frac{Admin\_unit\_prod}{Country\_prod}\times Country\_prod2014$$

2 $$Admin\_unit\_ha\_2014=\frac{Admin\_unit\_ha}{Country\_ha}\times Country\_ha2014$$

3 $$Prod\_percapita\_2014=\frac{Admin\_unit\_prod\_2014}{Admin\_unit\_pop2014}$$

4 $$HA\_percapita\_2014=\frac{Admin\_unit\_ha\_2014}{Admin\_unit\_pop2014}$$

A linear relationship between population number and cassava production was assumed up to a maximum allowed threshold value. The per capita cassava production and harvested area values were evenly allocated across all pixels by multiplying the number of people by the per capita value. It was assumed that a maximum of half of the area of the pixel can be allocated to cassava production (approx. 50 hectares) and the production per hectare does not exceed 20 tonnes per hectare. Thus, cassava harvested area per pixel cannot exceed 50 hectares and cassava production per pixel cannot exceed 1,000 tonnes. For those districts where cassava harvested area exceeded 50 hectares or production exceeded 1,000 tonnes in one or more pixels, the amount in excess was deducted and redistributed between the remaining pixels until the condition was satisfied and no pixel in a given administrative unit exceeded the assumed maximum production/harvested area threshold value.

## Data Records

CassavaMap provides representation of cassava production and harvested area in 32 countries of sub-Saharan Africa (Figs. 1 and 2). CassavaMap version 1.0 can be downloaded from the Figshare repository under CC BY 4.0 licence^23^. Data records constitute two raster TIFF files representing disaggregated cassava production (CassavaMap_Production_v1.tif) and harvested area (CassavaMap_HarvArea_v1.tif). CassavaMap_Prod_v1.csv and CassavaMap_HarvArea_v1.csv are data table files with an outline of detailed production and harvested area statistics in 474 and 644 administrative units respectively. Both files include information on source of cassava statistics data, administrative level, year of data records, rural population sum per administrative unit derived from LandScan 2014, summarised country production/harvested area, FAOSTAT production/harvested area per country in 2014, per capita production/harvested area, and per capita production/harvested area values adjusted to national production levels in 2014 as listed by FAOSTAT. CassavaMap_Legend.csv file explains the column headers for both data table files. Missing_data_input_calc.csv outlines the administrative units with missing data replaced by estimated values. The unique names or codes of neighbouring administrative units that were used to derive the new input per capita production or harvested area values are outlined in respective rows and the function used to estimate those values is listed. Newer versions of CassavaMap will appear in the repository as updated cassava production data become available.

## Technical Validation

CassavaMap represents a smooth fine-resolution disaggregation of cassava production and harvested area based on assumption that the distribution of rural population is the main driver of cassava density in sub-Saharan Africa. It is important to mention that model’s assumptions and its scale make it prone to under- or over-representation of cassava production if the underlying data are not accurate. First source of errors may be inherited from the LandScan 2014 population distribution model, which is based on a set of algorithms for redistributing population in given census zones. Second source of misrepresentation may occur where production statistics as reported by sources used in this study are over- or under-estimated. Finally, areas with coarse administrative granularity of data (especially these where data were available only on national scale), will have production/harvested area redistributed in a flatter manner underrepresenting regional variations in cassava planting intensity. Several areas of sharp contrast in cassava production or harvested area levels include Zambia/Malawi and Ghana/Burkina Faso borders.

CassavaMap v1.0 improves 10-fold the spatial resolution of the currently available crop distribution models and standardises the output to 2014 country-wide production levels reported by FAO.

## Data Availability

The data analysis was performed in R version 3.5.1^24^. The example of the custom code  used to disaggregate cassava production and harvested area data is publically available via GitHub at https://github.com/aniaszy/CassavaMap.

## Online-only Tables

**Online-only Table 1:** Sources of subnational cassava production data considered as a choice for the disaggregation analysis downloaded from: ReSAKSS (resakss.org), FAO Agro-MAPS (kids.fao.org/agromaps) and IITA (collection of data from various ministries of agriculture, personal correspondence with the International Institute of Tropical Agriculture in Ibadan, Nigeria).

Country	FAO Agro-Maps						IITA data with collated Ministries of Agriculture reported statistics						Resakss			
	Administrative level 1			Administrative level 2			2006 reported data		2007 reported data			2008 reported data				
	Year	Administrative units count	Administrative units with missing data count	Year	Administrative units count	Administrative units with missing data count	Administrative units count	Administrative units with missing data count	Administrative units count	Administrative units with missing data count	Administrative units count	Administrative units with missing data count	Year	Administrative units count	Administrative units with missing data count	Administrative level
Angola	2012	18	0	—	—	—	18	0	18	0	18	0	—	—	—	—
Benin	—	—	—	2009	77	6	77	1	77	1	77	1	2014	45	1	2
Burkina Faso	—	—	—	—	—	—	—	—	—	—	—	—	—	—	—	—
Burundi	2012	15	0	—	—	—	14	0	14	0	14	0	—	—	—	—
Cameroon	2007	10	1	2001	50	2	10	0	10	0	10	0	2010	10	0	1
CAR	1993	17	1	—	—	—	16	0	16	0	16	0	—	—	—	—
Chad	—	—	—	—	—	—	—	—	—	—	—	—	—	—	—	—
Congo	1990	9	2	—	—	—	5	0	5	0	5	0	—	—	—	—
Cote d’Ivoire	2007	19	0	—	—	—	19	0	19	0	—	—	2007	14	2	1
DRC	1996	11	0	1996	40	1	9	0	9	0	9	0	2014			1
Equatorial Guinea	—	—	—	—	—	—	—	—	—	—	—	—	—	—	—	—
Gabon	—	—	—	—	—	—	—	—	—	—	—	—	—	—	—	—
Gambia	—	—	—	—	—	—	—	—	—	—	—	—	—	—	—	—
Ghana	2011	11	2	—	—	—	10	2	10	2	10	2	2007–2011	137	9	2
Guinea	2001	8	5	1998	33	6	2	0	2	0	2	0	2015	7	0	1
Guinea—Bissau	2011	9	1	—	37	37	5	0	5	0	5	0	—	—	—	—
Kenya	2008	8	0	2000	47	19	45	2	—	—	—	—	2016	45	0	2
Liberia	1997	14	1	—	—	—	13	0	13	0	13	0	—	—	—	—
Malawi	2006	3	0	2006	28	2	24	0	24	0	—	—	2014	26	0	2
Mali	1996	8	7	1990	47	43	6	0	6	0	6	0	—	—	—	—
Mozambique	2008	10	0	2000	129	2	10	0	10	0	10	0	—	—	—	—
Niger	—	—	—	—	—	—	—	—	—	—	—	—	—	—	—	—
Nigeria	2005	37	0	—	—	—	37	6	37	1	37	5	2012	37	2	1
Rwanda	2000	12	1	—	—	—	11	0	11	0	11	0	2017	29	0	2
Senegal	—	—	—	—	—	—	—	—	—	—	—	—	—	—	—	—
Sierra Leone	—	—	—	—	—	—	—	—	—	—	—	—	—	—	—	—
South Sudan	—	—	—	—	—	—	—	—	—	—	—	—	—	—	—	—
Tanzania	2001	26	5	—	—	—	21	0	21	0	21	0	2014	21	0	1
Togo	2011	5	1	1994	21	8	6	1	6	1	6	1	2017	21	2	2
Uganda	2009	39*	0	—	—	—	39	3	39	3	39	3	2008	69	33	1
Zambia	2004	10	10	1992	58	2	8	0	8	0	8	0	—	—	—	—
Zimbabwe	—	—	—	—	—	—	—	—	—	—	—	—	—	—	—	—

^*^Corrupted polygon shapefile provided in the datasource.

**Online-only Table 2:** Sources of subnational cassava harvested area data considered as a choice for the disaggregation analysis downloaded from: ReSAKSS (resakss.org), FAO Agro-MAPS (kids.fao.org/agromaps) and IITA (collection of data from various ministries of agriculture, personal correspondence with the International Institute of Tropical Agriculture in Ibadan, Nigeria).

Country	FAO Agro-Maps						IITA data (collated Ministries of Agriculture reported statistics)						Resakss			
	Administrative level 1			Administrative level 2			2006		2007		2008					
	Year	Administrative units count	Missing data count	Year	Administrative units count	Missing data count	Administrative units count	Missing data count	Administrative units count	Missing data count	Administrative units count	Missing data count	Year	Administrative units count	Missing data count	Administrative level
Angola	2012	18	0	—	—	—	—	—	—	—	18	0	—	—	—	—
Benin	—	—	—	—	—	—	—	—	—	—	77	1	2017	77	0	2
Burkina Faso	—	—	—	—	—	—	—	—	—	—	—	—	—	—	—	—
Burundi	—	—	—	—	—	—	—	—	—	—	15	1	—	—	—	—
Cameroon	—	—	—	2001	50	12	—	—	—	—	10	0	2008	58	0	2
CAR	1993	17	1	—	—	—	—	—	—	—	17	1	2008	52	2	2
Chad	—	—	—	—	—	—	—	—	—	—	—	—	—	—	—	—
Congo	1990	9	2	—	—	—	—	—	—	—	9	4	—	—	—	—
Côte d’Ivoire	2007	69*	1	—	—	—	—	—	—	—	—	—	2007	14	2	1
DRC	1996	11	1	1996	40	1	—	—	—	—	9	0	—	—	—	—
Equatorial Guinea	—	—	—	—	—	—	—	—	—	—	—	—	—	—	—	—
Gabon	—	—	—	—	—	—	—	—	—	—	—	—	—	—	—	—
Gambia	—	—	—	2002	37	0	—	—	—	—	—	—	—	—	—	—
Ghana	2011	11	2	—	—	—	—	—	—	—	10	2	2013–2017	137	0	1
Guinea	2001	8	5	1998	33	11	—	—	—	—	2	0	2017	8	0	1
Guinea-Bissau	2011	9	1	1992	37	2	—	—	—	—	5	0	—	—	—	—
Kenya	2008	8	9	2000	47	25	—	—	—	—	—	—	2016	47	0	2
Liberia	1997	14	1	—	—	—	—	—	—	—	13	0	—	—	—	—
Malawi	2006	3	0	2006	28	4	—	—	—	—	—	—	2016	28	0	2
Mali	1996	8	2	1993	47	35	—	—	—	—	6	0	—	—	—	—
Mozambique	2008	10	1	2000	129	2	—	—	—	—	10	0	—	—	—	—
Niger	—	—	—	—	—	—	—	—	—	—	—	—	—	—	—	—
Nigeria	2005	38	7	—	—	—	—	—	35	2	—	—	2012	38	0	1
Rwanda	2000	12	1	2010	29	1	—	—	—	—	11	0	2017	30	0	2
Senegal	2007	10	0	1996	30	20	—	—	—	—	—	—	—	—	—	—
Sierra Leone	—	—	—	2004	14	0	—	—	—	—	—	—	—	—	—	—
South Sudan	—	—	—	—	—	—	—	—	—	—	—	—	—	—	—	—
Tanzania	2001	26	6	2005	129	19	—	—	—	—	21	0	—	—	—	—
Togo	—	—	—	1994	21	8	—	—	—	—	6	1	2017	37	0	2
Uganda	2009	39*	0	—	—	—	—	—	—	—	39	3	—	—	—	—
Zambia	—	—	—	1992	58	2	—	—	—	—	8	0	—	—	—	—
Zimbabwe	—	—	—	—	—	—	—	—	—	—	—	—	—	—	—	—

*Corrupted polygon shapefile provided in the datasource.

**Online-only Table 3:** Data sources for cassava production distribution model: FAO (fao.org/faostat), Resakss (resakss.org), FAO Agro-MAPS (kids.fao.org/agromaps) and IITA (personal correspondence with the International Institute of Tropical Agriculture). Cassava production statistics were standardised to reflect the country-wide totals reported for 2014 in FAOSTAT.

Country	Data source	Administrative level	Year	# adm units	Missing data
Angola	Agro-MAPS	1	2012	18	0
Benin	Agro-MAPS	2	2014	77	5
Burkina Faso	FAO	0	2014	1	0
Burundi	Agro-MAPS	1	2012	15	0
Cameroon	ReSAKSS	1	2010	10	0
CAR	IITA	1	2008	17	1
Chad	FAO	0	2014	1	0
Congo	Agro-MAPS	1	1990	9	2
Côte d’Ivoire	Agro-MAPS	1	2007	19	0
DRC	ReSAKSS	1	2015	11	0
Equatorial Guinea	FAO	0	2014	1	0
Gabon	FAO	0	2014	1	0
Gambia	FAO	0	2014	1	0
Ghana	Agro-MAPS	1	2011	11	2
Guinea	ReSAKSS	1	2015	8	1
Guinea-Bissau	FAO	0	2014	1	0
Kenya	Agro-MAPS	1	2008	8	0
Liberia	IITA	1	2008	14	1
Malawi	ReSAKSS	2	2014	28	0
Mali	IITA	1	2008	8	2
Mozambique	Agro-MAPS	1	2008	10	0
Niger	FAO	0	2014	1	0
Nigeria	IITA	1	2007	37	0
Rwanda	ReSAKSS	2	2017	30	0
Senegal	Agro-MAPS	1	1999	10	0
Sierra Leone	FAO	0	2014	1	0
South Sudan	FAO	0	2014	1	0
Tanzania	ReSAKSS	1	2014	30	5
Togo	Agro-MAPS	2	2011	5	1
Uganda	Agro-MAPS	1	2009	79	2
Zambia	IITA	1	2008	10	1
Zimbabwe	FAO	0	2014	1	0

**Online-only Table 4:** Data sources for cassava harvested area distribution model: FAO (fao.org/faostat), ReSAKSS (resakss.org), FAO Agro-MAPS (kids.fao.org/agromaps) and IITA (collection of data collectepersonal correspondence with the International Institute of Tropical Agriculture in Ibadan, Nigeria). Cassava harvested area statistics were standardised to reflect the country-wide totals reported for 2014 in FAOSTAT.

Country	Data source	Administrative level	Year	# adm units	Missing data
Angola	Agro-MAPS	1	2012	18	0
Benin	ReSAKSS	2	2017	76	0
Burkina Faso	FAO	0	2014	1	0
Burundi	IITA	1	2008	15	1
Cameroon	ReSAKSS	2	2008	52	2
CAR	IITA	1	2008	17	1
Chad	FAO	0	2014	1	0
Congo	IITA	1	2008	9	4
Côte d’Ivoire	Agro-MAPS	1	2007	19	0
DRC	IITA	1	2008	11	2
Equatorial Guinea	FAO	0	2014	1	0
Gabon	FAO	0	2014	1	0
Gambia	Agro-MAPS	2	1998	37	22
Ghana	Agro-MAPS	1	2007	11	2
Guinea	ReSAKSS	1	2017	8	1
Guinea-Bissau	IITA	1	2008	9	4
Kenya	ReSAKSS	2	2014	47	7
Liberia	IITA	1	2008	14	1
Malawi	ReSAKSS	2	2016	28	0
Mali	IITA	1	2008	8	2
Mozambique	Agro-MAPS	1	2007	10	1
Niger	FAO	0	2014	1	0
Nigeria	IITA	1	2007	37	2
Rwanda	ReSAKSS	2	2017	30	0
Senegal	Agro-MAPS	1	1998	10	0
Sierra Leone	Agro-MAPS	2	2004	14	0
South Sudan	FAO	0	2014	1	0
Tanzania	IITA	1	2008	26	6
Togo	ReSAKSS	2	2014	37	4
Uganda	Agro-MAPS	1	2009	79	2
Zambia	IITA	1	2008	9	1
Zimbabwe	FAO	0	2014	1	0

**Online-only Table 5:** Outline of administrative units (listed by region code) with missing data. Per capita production and harvested area data were derived calculating the average of the neighbouring administrative units outlined in the table.

Grid	AdmLevel	Year	Source	Country	Region_code	Adm_units	Function
CassavaMap_Production	2	2009	Agro-MAPS	Benin	BEN004999	BEN004006, BEN005004, BEN001084, BEN001641	Average
CassavaMap_Production	2	2009	Agro-MAPS	Benin	BEN007997	BEN005001, BEN001189, BEN001641, BEN007004, BEN002004, BEN002007, BEN004007, BEN001496	Average
CassavaMap_Production	2	2009	Agro-MAPS	Benin	BEN007998	BEN005001, BEN001189, BEN001641, BEN007004, BEN002004, BEN002007, BEN004007, BEN001496	Average
CassavaMap_Production	2	2009	Agro-MAPS	Benin	BEN007999	BEN005001, BEN001189, BEN001641, BEN007004, BEN002004, BEN002007, BEN004007, BEN001496	Average
CassavaMap_Production	1	1990	Agro-MAPS	Congo	COG003	COG006	Average
CassavaMap_Production	1	1990	Agro-MAPS	Congo	COG005	COG009, COG002	Average
CassavaMap_Production	1	2011	Agro-MAPS	Ghana	GHA008	GHA007, BFA001	Average
CassavaMap_Production	1	2011	Agro-MAPS	Ghana	GHA009	GHA007, BFA001	Average
CassavaMap_Production	1	2008	IITA	Liberia	LBR013	LBR014, LBR009, LBR006, LBR005	Average
CassavaMap_Production	1	2011	Agro-MAPS	Togo	TGO005	TOG002, BFA001	Average
CassavaMap_HarvArea	2	2014	Resakss	Kenya	Garissa	Samburu, Marsabit	Average
CassavaMap_HarvArea	2	2014	Resakss	Kenya	Isiolo	Samburu, Marsabit	Average
CassavaMap_HarvArea	2	2014	Resakss	Kenya	Kericho	Kiambu	Average
CassavaMap_HarvArea	2	2014	Resakss	Kenya	Mandera	Samburu, Marsabit	Average
CassavaMap_HarvArea	2	2014	Resakss	Kenya	Turkana	Samburu, Marsabit	Average
CassavaMap_HarvArea	2	2014	Resakss	Kenya	Wajir	Samburu, Marsabit	Average
CassavaMap_HarvArea	2	2014	Resakss	Kenya	West Pokot	Samburu, Marsabit	Average
CassavaMap_HarvArea	1	2009	Agro-MAPS	Uganda	UGA063	UGA064	Average
CassavaMap_HarvArea	1	2009	Agro-MAPS	Uganda	UGA102	UGA074	Average
CassavaMap_HarvArea	1	2007	IITA	Zambia	ZMB009	ZMB006	Average
CassavaMap_HarvArea	1	2008	IITA	Burundi	BDI009	BDI012, BDI13	Average
CassavaMap_HarvArea	1	2008	IITA	Congo	COG003	COG006	Average
CassavaMap_HarvArea	1	2008	IITA	Congo	COG005	COG009, COG002	Average
CassavaMap_HarvArea	1	2008	IITA	Congo	COG007	COG004, COG008, COG002	Average
CassavaMap_HarvArea	1	2008	IITA	Congo	COG009	COG016, COG002	Average
CassavaMap_HarvArea	1	2007	Agro-MAPS	Ghana	GHA008	GHA007	Average
CassavaMap_HarvArea	1	2007	Agro-MAPS	Ghana	GHA009	GHA007	Average
CassavaMap_HarvArea	1	2008	IITA	Guinea-Bissau	GNB008	GNB001	Average
CassavaMap_HarvArea	1	2008	IITA	Guinea-Bissau	GNB009	GNB006, GNB001	Average
CassavaMap_HarvArea	1	2008	IITA	Liberia	LBR013	LBR014, LBR009, LBR006, LBR005	Average
CassavaMap_HarvArea	1	2008	IITA	Mali	MLI001	MLI008	Average
CassavaMap_HarvArea	1	2008	IITA	Mali	MLI003	MLI008	Average
CassavaMap_HarvArea	1	2007	Agro-MAPS	Mozambique	MOZ001	MOZ007, MOZ006	Average
CassavaMap_HarvArea	1	2007	IITA	Nigeria	NGA018	NGA020, NGA021, NGA036, NGA005	Average
CassavaMap_HarvArea	1	2007	IITA	Nigeria	NGA037	NGA034, NGA021	Average
CassavaMap_HarvArea	1	2008	IITA	United Republic of Tanzania	TZA020	TZA026, TZA017, TZA013, TZA002	Average
CassavaMap_HarvArea	1	2007	IITA	Zambia	ZMB006	ZMB009, ZMB003	Average
